# Reduced neurite density index in the prefrontal cortex of adults with autism assessed using neurite orientation dispersion and density imaging

**DOI:** 10.3389/fneur.2023.1110883

**Published:** 2023-08-11

**Authors:** Takashi Arai, Koji Kamagata, Wataru Uchida, Christina Andica, Kaito Takabayashi, Yuya Saito, Rukeye Tuerxun, Zaimire Mahemuti, Yuichi Morita, Ryusuke Irie, Eiji Kirino, Shigeki Aoki

**Affiliations:** ^1^Department of Radiology, Juntendo University Graduate School of Medicine, Tokyo, Japan; ^2^Faculty of Health Data Science, Juntendo University, Chiba, Japan; ^3^Department of Radiology, Graduate School of Medicine, The University of Tokyo, Tokyo, Japan; ^4^Department of Psychiatry, Juntendo University Graduate School of Medicine, Tokyo, Japan; ^5^Department of Psychiatry, Juntendo University Shizuoka Hospital, Shizuoka, Japan

**Keywords:** autism, diffusion-weighted imaging, diffusion tensor imaging, gray matter, neurite orientation dispersion and density imaging, neuronal loss, surface-based cortical thickness measurement

## Abstract

**Background:**

Core symptoms of autism-spectrum disorder (ASD) have been associated with prefrontal cortex abnormalities. However, the mechanisms behind the observation remain incomplete, partially due to the challenges of modeling complex gray matter (GM) structures. This study aimed to identify GM microstructural alterations in adults with ASD using neurite orientation dispersion and density imaging (NODDI) and voxel-wise GM-based spatial statistics (GBSS) to reduce the partial volume effects from the white matter and cerebrospinal fluid.

**Materials and methods:**

A total of 48 right-handed participants were included, of which 22 had ASD (17 men; mean age, 34.42 ± 8.27 years) and 26 were typically developing (TD) individuals (14 men; mean age, 32.57 ± 9.62 years). The metrics of NODDI (neurite density index [NDI], orientation dispersion index [ODI], and isotropic volume fraction [ISOVF]) were compared between groups using GBSS. Diffusion tensor imaging (DTI) metrics and surface-based cortical thickness were also compared. The associations between magnetic resonance imaging-based measures and ASD-related scores, including ASD-spectrum quotient, empathizing quotient, and systemizing quotient were also assessed in the region of interest (ROI) analysis.

**Results:**

After controlling for age, sex, and intracranial volume, GBSS demonstrated significantly lower NDI in the ASD group than in the TD group in the left prefrontal cortex (caudal middle frontal, lateral orbitofrontal, pars orbitalis, pars triangularis, rostral middle frontal, and superior frontal region). In the ROI analysis of individuals with ASD, a significantly positive correlation was observed between the NDI in the left rostral middle frontal, superior frontal, and left frontal pole and empathizing quotient score. No significant between-group differences were observed in all DTI metrics, other NODDI (i.e., ODI and ISOVF) metrics, and cortical thickness.

**Conclusion:**

GBSS analysis was used to demonstrate the ability of NODDI metrics to detect GM microstructural alterations in adults with ASD, while no changes were detected using DTI and cortical thickness evaluation. Specifically, we observed a reduced neurite density index in the left prefrontal cortices associated with reduced empathic abilities.

## 1. Introduction

Autism-spectrum disorder (ASD) is a childhood-onset, lifelong neurodevelopmental disorder characterized by impaired social communication and social interaction and restricted, repetitive patterns of interest and behavior (1). Individuals with ASD are estimated to account for approximately 1% of the population (2). Social communication deficits, the core symptom of ASD, are associated with altered prefrontal cortex functions involving conceptual reasoning, imitation, language production, empathy, and social processing networks. Postmortem brain pathology studies in ASD have also reported a decrease in the number of microtubule-associated protein 2-expressed dendrites in the prefrontal cortex (3). ASD is also known to have genomic mutations in Src homology 3 and multiple ankyrin repeat domains 3 (SHANK3), a protein that is highly expressed in dendritic spines in the cerebral cortex and is an important scaffold protein that binds to and interacts with various receptors to form functional synapses. SHANK3 mutations are thought to be associated with behavioral abnormalities in ASD, such as a lack of social interaction (4–7). Studies of mouse models of ASD with SHANK3 mutations also have identified impaired amino-3-hydroxy-5-methyl-4-isoxazole-propionic acid receptor-mediated glutamatergic synaptic transmission and impaired dendritic spine remodeling (8). Thus, an association between synaptic and neuronal dysfunction in the gray matter (GM) and pathogenesis of ASD was suggested. However, the causal relationship between GM microstructural alterations and autistic traits is not fully understood, mainly due to the small number of autopsy samples. Furthermore, *in vivo* evidence of pathological changes in GM structures in patients with ASD should be further clarified. Complementary studies using animal models of ASD have attempted to fill the gap in human postmortem studies of ASD. Nevertheless, ASD is a heterogeneous disorder with multiple causes and different onset times; thus, a single genetically modified animal model cannot be expected to reproduce the full spectrum of ASD pathologies (9).

Recently, attempts have been made to characterize the microstructure of the autistic brain by studying the random motion of water molecules using diffusion tensor imaging (DTI), which can measure the orientation distribution of molecules, magnitude, and direction (orthogonal or perpendicular to the axon) of diffusion such as fractional anisotropy (FA), mean diffusivity (MD), and radial diffusivity (RD) (10–15). However, the microstructural alterations in the GM, particularly in adults with ASD, have not been widely explored. So, far, only one study reported reduced FA and increased MD in GM–white matter (WM) boundary in adults with ASD in comparison with typically developing (TD) individuals (16). To the best of our knowledge, no other report has used DTI to study diffusion quantitative values of gray matter only in adults with ASD. This might be partially due to the limitations of DTI. The model of Gaussian anisotropic diffusion in DTI is nonspecific for *in vivo* changes (17). In addition, partial volume effects of the cerebrospinal fluid (CSF) may affect DTI quantification in cortical areas (18). Thus, DTI is unsuitable for the assessment of GM regions, especially the cerebral cortex.

By contrast, neurite orientation dispersion and density imaging (NODDI) is a promising diffusion MRI (dMRI) technique for assessing GM. This diffusion model can be used to quantify the structural properties of neuronal projections such as axons and dendrites, which are challenging to estimate with DTI (19). NODDI is a biophysical tissue model that characterizes diffusion signals within each voxel into the following three compartments: intracellular, extracellular, and CSF. The NODDI model encompasses three measures, namely the neurite density index (NDI), orientation dispersion index (ODI), and isotropic volume fraction (ISOVF). These NODDI-derived measures act as indicators of dendritic density or the myelinated axon density, degree of dendritic dispersion, and CSF composition, respectively (20). The three-compartment model of NODDI can be used to reduce the influence of the partial volume effects on the complex cortical structure of the brain. Previous studies have indicated the usefulness of NODDI metrics as *in vivo* estimates of GM microstructural changes in major psychiatric disorders and some neurodegenerative diseases, including schizophrenia and Parkinson’s disease. A study on patients with schizophrenia showed reduced NDI in the temporal lobe cortex and significant associations between NDI and visuospatial working memory in various cortical areas of the frontal and temporal lobes (21). Furthermore, patients with Parkinson’s disease had reduced NDI in the substantia nigra pars compacta associated with disease duration and severity (22). Another study on Parkinson’s disease also suggested that NODDI may be sensitive to changes in GM microstructural alterations (23).

Although GM changes have been demonstrated in ASD, postmortem studies display significant heterogeneity due to various comorbidities (e.g., language impairment or intellectual disability). In addition, the broad age range of the postmortem sample makes it difficult to separate primary changes arising due to disability from secondary developmental effects. Herein, we aimed to investigate potential microstructural abnormalities in specific regions of the cerebral cortex in adults with ASD. We primarily focused on the prefrontal cortex, which is known to be associated with social communication deficits, a core manifestation of ASD. Previous pathological studies have reported cortical microstructural abnormalities in this region (3, 9, 24). Notably, a recent postmortem examination of adult autism cases revealed a novel cellular architectural abnormality known as “pencil fiber” (18). Considering the possibility of distinctive microstructural abnormalities in the prefrontal cortex of adults with ASD, we aimed to explore the utility of NODDI for non-invasive assessments of gray matter microstructures *in vivo*. However, to the best of our knowledge, reports analyzing gray matter in adults with ASD using NODDI are limited.

Several multi-shell dMRI studies on GM in ASD have been reported thus far; a study using Restriction Spectrum Imaging (RSI) (25) was possibly the first to report the first multi-shell diffusion model in autistic GM. RSI extends the spherical deconvolution model across multiple length scales and characterizes neurite density and organization at each imaged voxel. Subjects with ASD aged 7–17 years were studied, and diffusion quantiles were measured in 48 cortical regions of interest (ROI) per hemisphere. Using RSI, Carper et al. indicated GM microstructural changes (indexed by lowered intra-axonal diffusivity) in the anterior cingulate, right superior temporal lobe, and much of the parietal lobe regions of ASD compared with TD patients; however, the intergroup differences were not significant. A study using the NODDI (26) examined the relationship between the NODDI index and the facial expression recognition performance in GM voxels using a voxel-based analysis (VBA) approach in ASD subjects aged 18–25 years. The results suggested that ASD patients have deficits in the perceptual integration of facial expressions across the cerebral hemispheres. However, VBA is largely affected by partial volume effects, especially in the subcortical WM of the sulci, due to spatial smoothing that excludes individual differences in brain structures. Consequently, VBA is inaccurate in fitting the brain anatomy to a common template across individuals, especially for those with local brain morphological changes. A study using diffusion kurtosis imaging (27) found significant reductions in kurtosis in the frontal lobes, temporal lobes, and some of the sublobar regions associated with the pathophysiology of ASD. The reduction in kurtosis in the autism group was also correlated with symptoms of autism, suggesting that reduced kurtosis is a measure associated with GM abnormalities (reduced neuronal density and size, altered cortical column size, and restricted dendrites) in the postmortem pathology of autistic brains and that it is also associated with symptoms of autism that were considered pathological. Recently, a study of ASD GM using the NODDI and GBSS framework was reported (28) in which diffusion-quantified values of the GM microstructure in ASD and TD subjects aged 5–21 years as a comparison were analyzed on a voxel-by-voxel basis. The relationship between age-related difference and between-group difference was also examined. Results showed that the NDI was significantly increased in the frontal, temporal, and occipital regions of the right hemisphere in ASD subjects. Neurite dispersion correlated with autistic traits. The results suggest that neurodevelopmental changes in autism affect the GM microstructure and that the cortical microstructure plays a role in symptoms of autism. Matsuoka K et al. observed significantly higher ODIs within the left occipital gyrus, which corresponded to the secondary visual cortex, in individuals with ASD. Furthermore, they observed a significant negative correlation between the functional connectivity values of the left occipital and left middle temporal gyri and the mean ODI values. These findings indicated that alterations in dendritic complexity within the left occipital gyrus might be linked to changes in distal connectivity with the left middle temporal gyrus in ASD (22). In another study, Kitamura S et al. reported a positive correlation between NDI and scores representing hypersensitivity or sensory sensitivity in the right superior temporal gyrus in individuals with ASD compared with normally developing individuals. Furthermore, NDI in the right superior temporal gyrus showed a significant increase in individuals with ASD who experienced severe adverse events during childhood compared with those with relatively milder adverse events. These findings indicated that cortical microstructural abnormalities, specifically increased NDI in the right superior temporal gyrus, play a significant role in the neural mechanisms underlying hypersensitivity to external stimulation in ASD (23). Using a surface-based analytic approach, Cabana JF et al. observed significant reductions in MD and ISOVF, along with increased FA, primarily in the right hemisphere network among individuals with the SYN1Q555x mutation, which is associated with language disorders, epilepsy, and ASD (24). Currently, only one study has analyzed diffusion quantities using NODDI and GBSS in the cortex of patients with ASD, primarily involving subjects from childhood to adolescence (21). Therefore, changes in diffusion quantiles that would support the pathological changes observed in adult ASD GM postmortem remain largely unknown.

In this study, we proposed the use of GM-based spatial statistics (GBSS) analysis to reduce partial volume effects from the subcortical WM and CSF (29) in adults with ASD. GBSS is designed to analyze the GM using a skeleton projection step to ensure accurate anatomical alignment between individuals (30). Unlike VBA, GBSS can avoid smoothing problems and provide more robust between-group comparisons.

In this study, we aimed to assess GM microstructural alterations in adults with ASD using NODDI and GBSS and its association with comprehensive autism-related tests, such as autism-spectrum quotient (AQ), empathizing quotient (EQ), and systemizing quotient (SQ) and evaluate DTI metrics and surface-based cortical thickness (SBCT) for comparison.

## 2. Materials and methods

### 2.1. Study participants

The Research Ethics Committee of Juntendo University Hospital in Tokyo, Japan, approved the study protocol, and written informed consent was obtained from all study participants. Individuals with ASD were recruited from the outpatient clinics of Juntendo Koshigaya Hospital (Saitama, Japan) and Juntendo Shizuoka Hospital (Shizuoka, Japan). TD participants were recruited from the staff at the same hospitals and were selected so that they would be matched with the ASD group in terms of age, sex, and years of education. A total of 48 participants were included, of which 22 had ASD (17 men; mean age, 34.42 ± 8.27 years) and 26 were TD individuals (14 men; mean age, 32.57 ± 9.62 years). All participants were right-handed. Table 1 shows the demographic and clinical characteristics of the study participants.

**Table 1:** Demographic and clinical characteristics of the participants.

	TD (*N* = 26)	ASD (*N* = 22)	*p* value
Sex (N, male/female)	17/9	14/8	0.900
Age (mean ± SD; years)	34.42 ± 8.27	32.57 ± 9.62	0.482
Years of education (mean ± SD)	15.11 ± 2.53	14.88 ± 2.03	0.732
ICV (mean ± SD; mL)	1343.52 ± 298.68	1415.81 ± 233.14	0.363
Average absolute motion (mean ± SD; mm)	0.71 ± 0.61	0.68 ± 0.29	0.812
Average relative motion (mean ± SD; deg)	0.52 ± 0.48	0.59 ± 0.14	0.484
Clinical scores (mean ± SD)			
AQ-total	15.88 ± 6.13	32.13 ± 5.85	**<0.001**
AQ-social skills	2.28 ± 2.03	6.63 ± 1.55	**<0.001**
AQ-attention switching	3.56 ± 1.78	7.18 ± 2.06	**<0.001**
AQ-attention to detail	4.76 ± 2.12	5.36 ± 1.98	0.365
AQ-communication skills	1.92 ± 1.84	7.00 ± 2.02	**<0.001**
AQ-imagination	3.36 ± 1.72	5.95 ± 1.73	**<0.001**
SQ	24.76 ± 11.44	26.68 ± 13.43	0.765
EQ	36.08 ± 9.41	26.40 ± 7.06	**<0.001**

Bold values denote statistical significance at *p* < 0.05. ASD, autism-spectrum disorder; AQ, autism-spectrum quotient; EQ, emotional quotient; ICV, intracranial volume; SQ, systemizing quotient; TD, typically developing.

**Figure 1:**
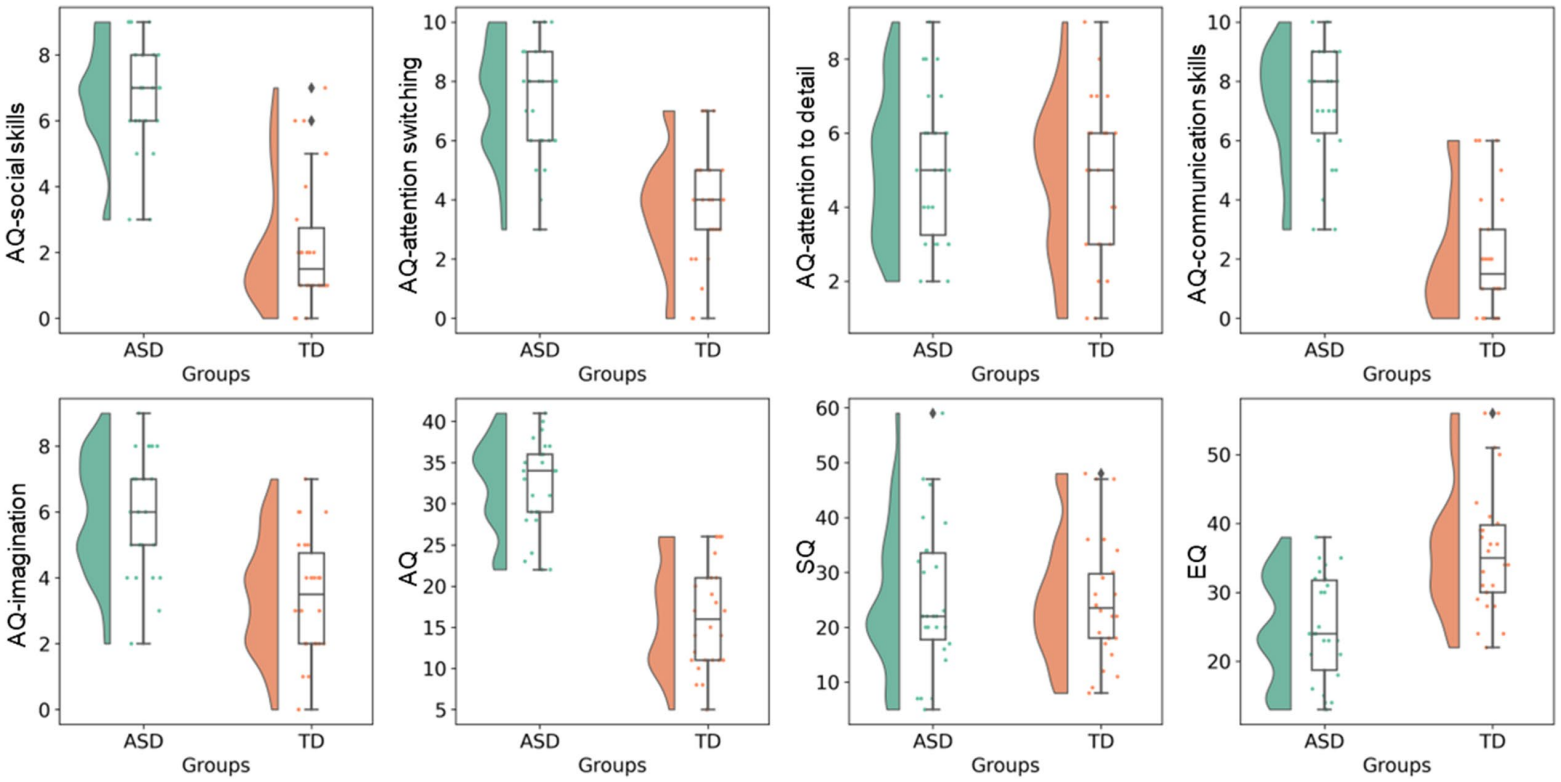
ASD-related clinical scores. Raincloud plots of ASD-related clinical scores (i.e., total AQ, AQ subscales, EQ, and SQ) are shown for the ASD and TD groups. AQ, Autism-spectrum quotient; ASD, autism-spectrum disorder; EQ, emotional quotient; SQ, Systemizing quotient; TD, typically developing.

ASD was diagnosed based on the fifth edition of the *Diagnostic and Statistical Manual of Mental Disorders* (31). TD participants did not have a history of any psychiatric, neurologic, or developmental disorders. None of the participants reported a history of head injury. Each participant was assessed using AQ (32), EQ (33), and SQ (34). AQ, EQ, and SQ are self-administered measures (for use with adults of normal intelligence) for autistic traits, social functioning, and the capability to analyze or construct systems, respectively. AQ comprises five subscales (social skills, attention switching, attention to detail, communication skills, and imagination) and represents the degree to which a person shows autistic traits (the higher the score, the higher the degrees of autistic traits). EQ reports the level of empathy (the lower the score, the lower the empathizing skills, which are responsible for the social difficulties associated with ASD). SQ is an indicator of the strength of interest in systematization. Raincloud plots of ASD-related clinical scores for the ASD and TD groups are shown in Figure 1.

### 2.2. MRI data acquisition

All MRI data, including diffusion-weighted images (DWI) and three-dimensional (3D) T1-weighted images were acquired on a 3-T Achieva scanner (Philips Healthcare, Best, Netherlands). Multishell diffusion-weighted imaging was performed with the following sequence parameters: b-values, 1,000 and 2000 s/mm^2^; 32 diffusion-weighted directions (both b-values = 1,000 and 2000 s/mm^2^); one no-gradient weighted image (b0); echo time [TE], 100 ms; repetition time [TR], 9,810 ms; flip angle, 90°; matrix size, 128 × 128; field of view [FOV], 256 × 256 mm; slice thickness, 2 mm; and acquisition time, 13 min 7 s. The sequence parameters of T1WI were as follows: TE, 3.4 ms; TR, 15 ms; inversion time, 932 ms; flip angle, 10°; matrix size, 256 × 256; FOV, 256 × 256 mm; slice thickness, 1 mm; turbo field echo factor, 116; shot interval time, 2,500 ms, and acquisition time, 5 min 14 s.

### 2.3. Diffusion-weighted imaging processing

All data sets were visually checked in all three orthogonal (axial, sagittal, and coronal) views, and none showed any severe artifacts, such as gross geometric distortion, signal dropout, and bulk motion. Diffusion-weighted data were subsequently corrected for susceptibility-induced geometric distortion, eddy current distortion, and intervolume subject motion using the EDDY (35). In diffusion imaging studies of ASD, sufficiently focusing on quality control is crucial (36). Quality control metrics related to volume-to-volume motion at the EDDY step, such as average absolute motion (mm) and average relative motion (degree), were compared between the ASD and TD groups; however, no differences were observed.

**Figure 2:**
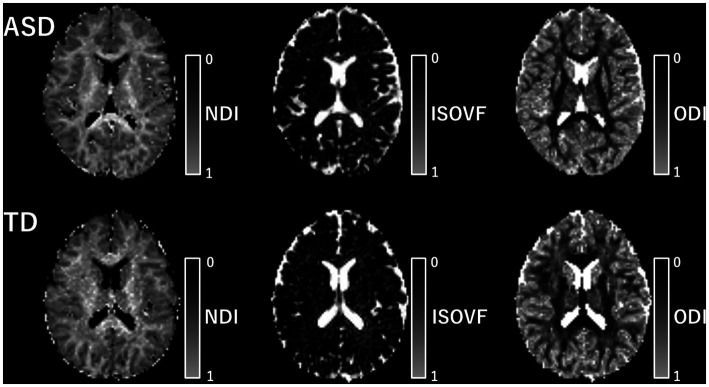
Map of each NODDI metric. The map of each NODDI metric is shown. The upper row includes the metric of patients with ASD and the lower row includes that of TD individuals. ASD, autism-spectrum disorder; ISOVF, isotropic volume fraction; NDI, neurite density index; ODI, orientation dispersion index; TD, typically developing.

In this study, we utilized the accelerated microstructure imaging via convex optimization (AMICO) technique (37) instead of the conventional NODDI toolbox to obtain NODDI-derived metrics (NDI, ODI, and ISOVF) for each voxel across all subjects, resulting in improved processing time. Previous reports have demonstrated that using AMICO does not compromise the accuracy and robustness of the estimated parameters (37). The AMICO script is available online.^1^ The diffusion tensor was estimated using the ordinary least-squares method applied to DWI at b = 0 and 1,000 s/mm^2^. FA, MD, AD, and RD maps were then produced based on standard formulas (38). A mask consisting of all brain voxels with an ISOVF <90% was applied to all diffusion parameter maps to reduce the influence of the partial volume effects of CSF (39). The intrinsic diffusivity for NODDI was assumed to be 1.7 μm^2^·ms^−1^, which is the standard setting for the human brain (40). Maps of each NODDI indicator are shown in Figure 2.

### 2.4. GBSS analysis

DTI (FA, MD, AD, and RD) and NODDI (NDI, ODI, and ISOVF) metrics were regionally compared between the ASD and TD groups using GBSS (29). GBSS is an analog pipeline of tract-based spatial statistics (TBSS) (41) to perform voxel-wise statistical analyzes on GM. GBSS applies a skeleton projection step to ensure accurate anatomical alignment between participants. The skeleton projection step of GBSS can reduce the effect of spatial misalignment along individuals due to complex cortical geometry and pathological features. Furthermore, GBSS can minimize the partial volume effect of the WM and CSF by aggregating the parameters of surrounding regions into those of the skeleton. In this study, we opted for a GBSS-based framework instead of a surface-based analysis like Freesurfer. The application of a smoothing kernel in the statistical pretreatment of surface-based analyzes with Freesurfer can result in misalignment of the measured cortical regions. Conversely, the GBSS-based framework used in this study provides the advantage of eliminating the requirement for smoothing. Furthermore, concerns were raised regarding the extensive computational time needed for cortical surface reconstruction with Freesurfer, as well as the necessity for rigorous quality control evaluation in surface-based analyzes (42).

GBSS was performed using the FMRIB Software Library version 6.0.3 (FSL) (43). The GBSS pipeline script is available online.^2^ Using GBSS, we analyzed GM as in previous studies (39, 43, 44) in accordance with the following procedure. First, we removed the skull from the 3D-T1WI volumes of each participant to exclude voxels other than the brain. Second, we affined and aligned the skull-stripped 3D-T1WI to the common space (MNI152 brain at 1-mm resolution) using FMRIB’s linear image registration tool and FMRIB’s nonlinear image registration, respectively. Third, the 3D-T1WI of each participant was bias corrected and segmented into three tissue classes (GM, WM, and CSF) using FMRIB’s automated segmentation tool. A mean GM map was created by merging GM probability images of all subjects and skeletonized to keep the core likely in the center of the GM (mean GM skeleton). We created the mean GM skeleton mask from the mean GM skeleton with a threshold of 0.2. The GM skeleton distance map was created from the mean GM image, and the GM skeleton mask was drawn using the “distancemap” program implemented in FSL. Fourth, after the b0 map of each participant was aligned with 3D-T1WI (epi-reg), the diffusion parameter maps of the participants were affined and nonlinearly aligned into a common space (MNI152 brain at 1-mm resolution). Finally, we projected the aligned diffusion parameter maps for each participant onto the mean GM skeleton map.

### 2.5. ROI analysis

The GM regions were automatically defined using the Desika–Killiany atlas implemented in FreeSurfer^3^ and superimposed onto the GM skeleton of each subject. We then measured the mean DTI (FA, MD, RD, and AD) and NODDI (NDI, ODI, and ISOVF) values for each participant in the 18 skeletonized GM regions, including the left and right caudal middle frontal, lateral orbitofrontal, medial orbitofrontal, pars opercularis, pars orbitalis, pars triangularis, rostral middle frontal, superior frontal, and frontal pole regions. These regions are known to be associated with the pathophysiology of ASD. The prefrontal cortex is a region of the autistic brain in which previous pathology reports have reported cortical microstructural abnormalities. For example, ill-defined cortical layers, substantially depleted MAP2 neuronal expression, and reduction in neuronal dendrites have been reported in the dorsolateral prefrontal cortex of the autistic brain (3). Disruption of cortical cell structure and formation of pencil fibers, possibly due to cortical dysplasia, has also been reported in the prefrontal cortex of the autistic brain (24). In the present study, we conducted a correlation analysis in the prefrontal cortex based on the hypothesis that there is an association between NODDI quantitative values, which reflect microstructural changes, and clinical autistic characteristics.

### 2.6. Surface-based cortical thickness analysis

We performed SBCT analyzes using FreeSurfer (Fischl, B. (2012). FreeSurfer. Neuroimage 62, 774–781.) version 5.0.0 (Massachusetts General Hospital, Harvard Medical School; available at http://surfer.nmr.mgh.harvard.edu). The implemented processing pipeline involved nonbrain tissue removal, transformation to Talairach reference space, segmentation of subcortical WM and deep GM volume structures, intensity normalization, tessellation of GM–WM boundaries, automatic topology correction, and surface deformation according to intensity gradients. The GM–WM and GM–CSF boundaries could be also optimally placed where there was the largest intensity shift defining the transition to other tissue classes. The misclassification of tissue types was corrected with minimal manual adjustment by one of the authors (TA) who was blind to the clinical information of the participants. Cortical thickness was calculated as the shortest distance between the GM–WM boundary and the corresponding pial surface at each vertex across the cortical mantle. The maps were smoothed using a surface-based Gaussian kernel of 15-mm FWHM and averaged across participants. In addition, the intracranial volume (ICV), total GM volume, and total WM volume were calculated from the FreeSurfer processing stream. These volumes were extracted from the aseg.stats file, which is the output of FreeSurfer’s recon-all pipeline (refer to https://surfer.nmr.mgh.harvard.edu/fswiki/MorphometryStats).

### 2.7. Statistical analysis

Mann–Whitney U tests were used to compare age, years of education, ICV, AQ (total score and subscales), EQ, and SQ scores. The chi-square test was used to compare the individuals according to their sex between the ASD and TD groups. A *p* value of <0.05 was considered significant. These tests were conducted using IBM SPSS Statistics for Windows version 27 (IBM Corp., Armonk, NY, United States).

For GBSS analysis, the Randomize tool (45) of FSL version 6.0.3 was used to apply a family-wise error (FWE)-corrected *p* value to each cluster of voxels comprising the GM skeleton. The threshold-free cluster enhancement option was then used to avoid arbitrary cluster formation threshold selection by generating 10,000 permutations to provide an empirical null distribution for the maximum cluster size (39). Using the Randomize tool with nonparametric permutation tests, voxel-wise statistical analyzes between the ASD and TD groups were tested in each of DTI (FA, MD, AD, and RD) and NODDI (NDI, ODI, and ISOVF) metrics using a general linear model (GLM) framework with unpaired t-test while controlling for age, sex, and ICV. Between-group differences were considered significant at pFWE < 0.05.

For the ROI analysis, the associations between DTI (FA, MD, AD, and RD) or NODDI (NDI, ODI, and ISOVF) and ASD-related clinical scores (i.e., total AQ, AQ subscales, EQ, and SQ) were evaluated using partial correlation test with age, sex, and ICV included as confounding factors in SPSS version 27. The correlation analysis was performed separately in the ASD and TD groups. Bonferroni correction was used to correct multiple comparisons (seven different diffusion metrics, i.e., FA, MD, AD, RD, NDI, ODI, and ISOVF) with the threshold for statistical significance set at *p* < 0.05/7 = 0.0071. Multiple linear regression (MLR) with backward elimination was used to further examine the associations between diffusion MRI-based measures (dependent variables) and clinical scores (independent variables: total AQ, AQ subscales, EQ, and SQ scores). Age, sex, and ICV were incorporated as confounding factors in the analysis. To ensure the absence of multicollinearity among the predictor variables, the variance inflation factor (VIF) values were assessed.

For SBCT analysis, cortical thickness was compared between groups at each vertex of the surface using a GLM while controlling for age, sex, and ICV. The associations between cortical thickness and ASD-related clinical scores (i.e., total AQ, AQ subscales, EQ, and SQ) were also modeled while controlling for age, sex, and ICV. In all vertex-wise analyzes, the statistical significance level was evaluated using a cluster-wise *P* (CWP). The correction procedure for multiple comparisons was performed using the cluster-based Monte Carlo simulation with 10,000 permutations. Clusters with CWP value <0.05 were considered statistically significant.

## 3. Results

### 3.1. Study participants

Age, sex, years of education, ICV, average absolute motion, average relative motion, AQ-attention to detail score, and SQ score were not significantly different between the ASD and TD groups. The ASD group had significantly higher total AQ, AQ-social skills, AQ-attention switching, AQ-communication skills, and AQ-imagination scores and lower EQ score than the TD group (Table 1).

### 3.2. GBSS analysis

**Figure 3:**
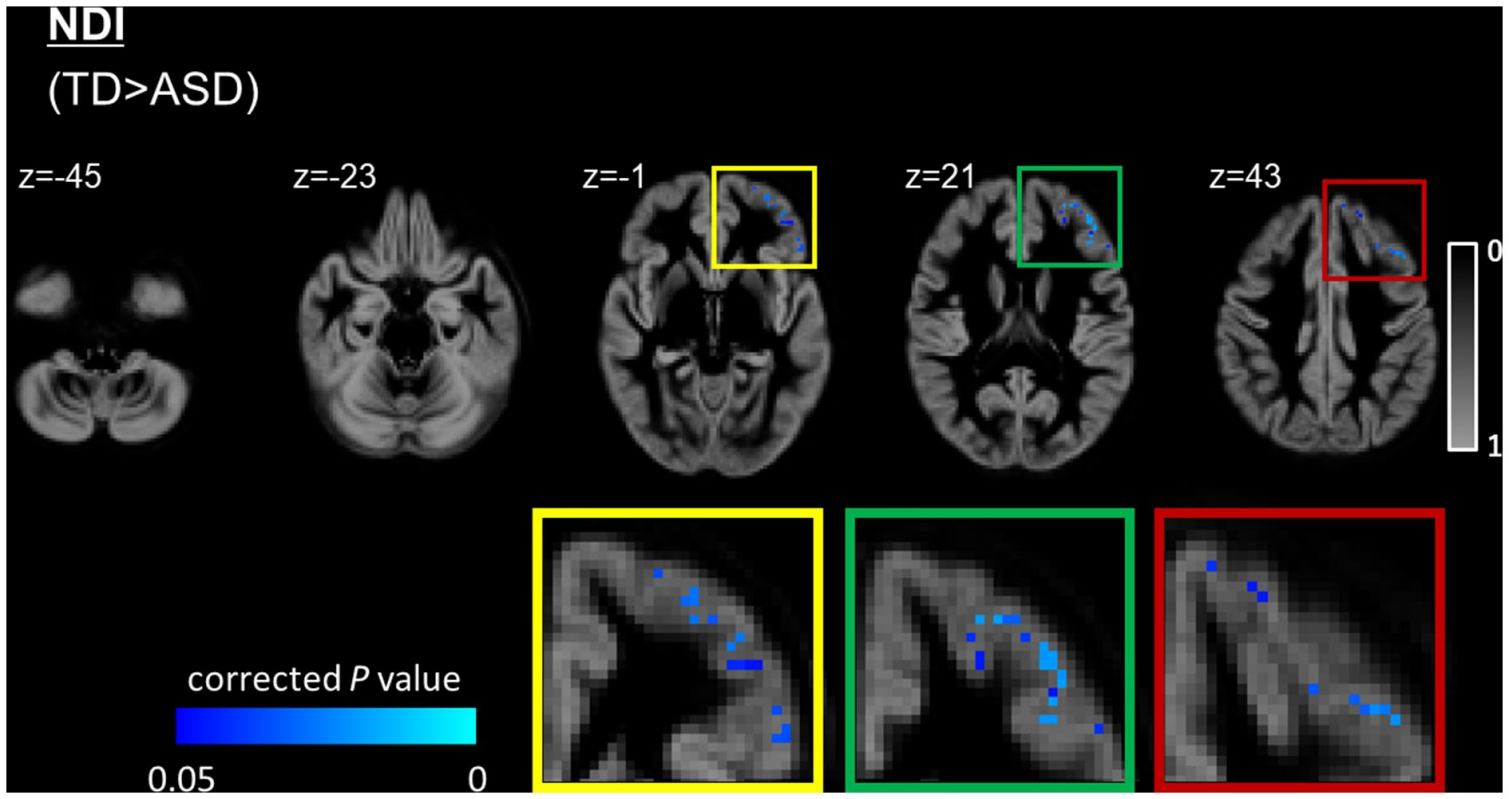
GBSS analysis of NDI between the ASD and TD groups. GM voxels are projected on the group-specific GM probability map (range 0–1). Significantly (FWE-corrected *p* < 0.05) lower (blue-light blue voxels) NDI is observed in the left prefrontal cortex, including the caudal middle frontal, lateral orbitofrontal, parsorbitalis, pars triangularis, rostral middle frontal, and superior frontal regions in the ASD group compared with the TD group. To aid visualization, results are thickened using the fill script implemented in FMRIB Software Library. ASD, autism-spectrum disorder; FWE, family-wise error; GBSS, gray matter-based spatial statistics; NDI, neurite density index; NODDI, neurite orientation dispersion and density imaging; TD, typically developing.

The ASD group had significantly (FWE-corrected *p* < 0.05) lower NDI than the TD group in the left caudal middle frontal, left lateral orbitofrontal, left pars orbitalis, left pars triangularis, left rostral middle frontal, and left superior frontal regions (Figure 3 and Table 2). No significant differences in FA, RD, AD, MD, ODI, and ISOVF were found between the groups.

**Table 2:** GBSS analysis of NDI in individuals with ASD and TD participants.

Modality	Cluster size	Anatomical regions	Peak *T*-value	Peak MNI coordinates
NDI (TD > ASD)	568	Left parts of the caudal middle frontal, lateral orbitofrontal, pars orbitalis, pars triangularis, rostral middle frontal, and superior frontal regions	4	X, 60; Y, 92; Z, 37

ASD, autism-spectrum disorder; GBSS, gray matter-based spatial statistics; NDI, neurite density index; TD, typically developing.

### 3.3. ROI analysis

**Figure 4:**
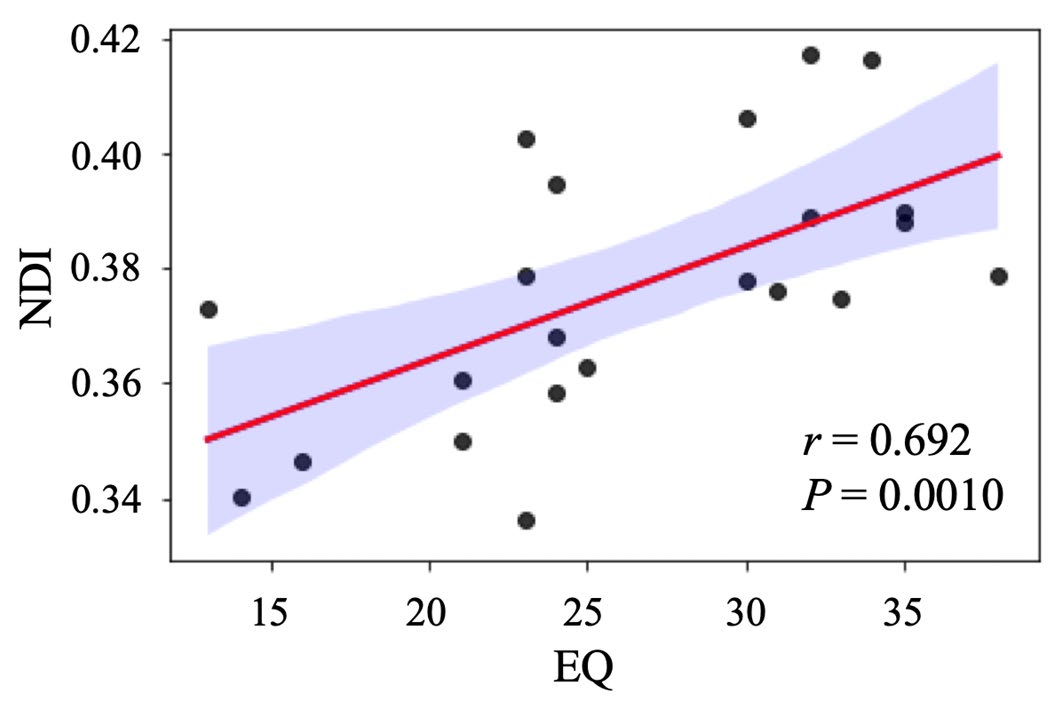
Correlation analysis between NDI and EQ in individuals with ASD. The scatter plot shows a significant positive correlation between NDI in the left rostral middle frontal gyrus and EQ after adjusting for age, sex, and intracranial volume. ASD, autism-spectrum disorder; EQ, emotional quotient; NDI, neurite density index.

Because we conducted multiple comparisons using seven different diffusion indices, we used a significant *p* value of <0.05/7 with the Bonferroni correction. In the ASD group, we observed a significant (*p* = 0.0010, *r* = 0.692; Figure 4) positive correlation between the NDI and EQ scores in the left rostral middle frontal region. In contrast, no significant (*p* = 0.871, *r* = 0.037) correlation was found between the NDI and EQ scores in the TD group. The MLR analysis further demonstrated the relationship between NDI and EQ scores in the left rostral middle frontal (standardized β = 0.47 [standard error: 0.00045], *p* = 0.027), left superior frontal (standardized β = 0.44 [standard error: 0.00045], *p* = 0.024), and left frontal pole (standardized β = 0.40 [standard error: 0.00071], *p* = 0.043), whereas a higher EQ score was associated with lower NDI. All independent variables had VIF values of <10, indicating the absence of multicollinearity. No significant associations were noted between NDI and EQ scores in other regions and between DTI (FA, MD, AD, and RD) or other NODDI (ODI and ISOVF) metrics and ASD-related clinical scores in the ASD or TD groups.

### 3.4. SBCT analysis

No significant differences in brain volumes or cortical thickness were found between the ASD group and the TD group. Moreover, no significant correlations were noted between brain volumes or cortical thickness and ASD-related clinical scores in the ASD or TD groups.

## 4. Discussion

In this study, GBSS analysis demonstrated a reduction in NDI predominantly in the left prefrontal cortices, including caudal middle frontal, lateral orbitofrontal, pars orbitalis, pars triangularis, rostral middle frontal, and superior frontal regions, in the ASD group when compared with the TD group. We also observed an association between reduced NDI and difficulties in empathy in the left rostral middle frontal cortex of the ASD group. Using partial correlation and MLR with backward elimination analyzes, we also observed an association between reduced NDI and difficulties in empathy in the left prefrontal cortices (i.e., rostral middle frontal, left superior frontal, and left frontal pole) in the ASD group.

ASD may be associated with the dysfunction of cortical regions, particularly the prefrontal cortex, a region associated with social communication deficits, the core symptom of ASD. Despite the lack of studies in adults with ASD, previous structural and functional neuroimaging studies have reported frontal lobe cortex abnormalities in children with ASD (46–48). In addition, an existing report on the GM of young ASD patients (5–21 years old, mean age 12.11 years) using GBSS reported decreased NDI in the right frontal, right temporal, and right occipital lobes (28). In this study, our findings provide further evidence that disruption of cortical cytoarchitecture in the prefrontal cortex is the neural basis for ASD in adults. A previous postmortem study showed significantly smaller pyramidal neurons and reduced dendritic density in the prefrontal cortex in ASD (9). Pyramidal neurons are the major excitatory neurons in the cortex and release glutamate at synapses of axon terminals to excite the following neurons and induce action potentials. Mutations in the chromodomain-helicase-DNA binding protein 8 gene, one of the most commonly reported genetic mutations in ASD, have also been reported to inhibit axonal and dendritic growth (49). In addition, other existing autism pathology reports have indicated that cortical cellular structures are disrupted in the prefrontal cortex and that pencil fibers are formed. It has been suggested that the formation of pencil fibers is a structural change resulting from abnormal cortical formation in autism (24). Our findings provide further evidence that disruption of cortical cytoarchitecture in the prefrontal cortex is the neural basis for ASD in adults. Furthermore, recent studies using DTI and NODDI have consistently shown microstructural changes (i.e., reduced FA and NDI and increased MD) in the anterior thalamic radiation (ATR) (50, 51). The ATR is a projection fiber connecting the dorsomedial nucleus of the thalamus to the prefrontal cortex, a WM tract functionally related to language and social processing and influencing communication. Considering these previous reports, further longitudinal studies are warranted to clarify whether WM alterations are one of the substrates responsible for disruption of cortical cytoarchitecture via corticocortical disconnection or vice versa.

Furthermore, the results of the present study demonstrated a significant association between reduced NDI in the rostral middle frontal, superior frontal, and left frontal pole and the lack of empathy in patients with ASD. Empathy is the ability to understand other people’s feelings and react emotionally to others, incorporating cognitive and emotional components. Individuals with ASD reportedly exhibit atypical empathic responses, which limit communication and social interactions (52). Similar to the findings reported in this study, the medial prefrontal cortex was reported to mediate human empathy (53). Furthermore, previous functional MRI studies have reported weaker interregional functional connectivity between the prefrontal cortex and the posterior cingulate gyrus, which are both brain regions involved in social behavior, in individuals with ASD (54).

The ASD group only showed reduced NDI in the left prefrontal cortex. This might suggest a tendency toward left–right asymmetry in cortical microstructural changes in ASD. Since the study participants with ASD were all right-handed, it may be associated with abnormal brain function in the dominant hemisphere. Indeed, left–right differences in the cerebrum have been reported several times in ASD. In line with our findings, functional connectivity was reported weaker in the dominant hemisphere in the autistic group than in the TD group during an irony comprehension task (55). Furthermore, left hemisphere regions are critical for language functions, especially in right-handed individuals (56). Previous MRI studies have indicated that the ASD group had a smaller left hemisphere, which impacts language development (57), and reported an association between reduced NDI and AQ-communication scores in the left ATR, superior longitudinal fasciculus, and uncinate fasciculus (51).

We also demonstrated that a voxel-wise GM analysis with GBSS enables the regional mapping and characterization of GM pathology in adults with ASD. Generally, the partial volume effects of the WM and CSF adjacent to the cortical GM produce some difficulties in obtaining accurate measurements of the cortical GM, a thin structure (a few mm) with complicated convolutional patterns. The GBSS method used in this study was developed based on the modification of the VBA for cortical analyzes, by aggregating the parameters of regions surrounding a skeleton created at the center of the cortical GM, thereby minimizing the PVE of the WM and CSF (30).

This study showed no significant differences in DTI metrics and cortical thickness between the ASD and TD groups. As aforementioned, DTI is not a common method for evaluating the GM (particularly in the cortex) because detecting the anisotropy of GM anatomy is challenging, which limits the widespread use of standard diffusion anisotropy metrics (58, 59). Meanwhile, cortical microstructural alterations were reported to precede morphological changes (60). Taken together, this finding lends further support to the suggestion that NODDI indices are potentially more sensitive as biomarkers for detecting cortical microstructural changes in ASD.

This study has several limitations. First, this cross-sectional study included a relatively small sample size from a single center, which might have limited the statistical power of the results and led to false-negative findings. Therefore, originally significant results could have been obtained in a wider GM area. The sample size in this study (26 ASD vs. 22 TD subjects) is comparable with that used in previous dMRI studies in ASD, for example, 26 ASD versus 25 TD participants (51) and 15 ASD versus 15 TD participants (61). Further studies should be conducted with a larger population from numerous centers and longitudinal data to establish evidence of ASD-associated GM neurodegeneration using NODDI. Second, since this study was conducted in adults with ASD, studies in children are needed to confirm whether NODDI metrics are effective as disease-specific biomarkers. Third, sufficient caution should be exercised when interpreting that NODDI can quantify brain microstructure in the current context. For example, a study reported that NDI, a measure of NODDI, was not significantly associated with the fraction of astrocytes and microglia that compose the extracellular space of neurons (58). NODDI does not take into account the structural characteristics of each brain region because it uses a fixed value for intra-axial diffusivity in any region of the brain. Additionally, NODDI does not adequately reflect both dendritic and axonal density. Water diffusion in axons and dendrites have different characteristics, and quantitative diffusion values were reported to be associated with myelinated axons but not with dendrites (59). In the present study, we utilized NODDI to improve the specificity of the observed GM microstructural changes in adults with ASD. Nevertheless, interpreting the results with caution is crucial considering that the NODDI model still needs further validation. Fourth, the neurite compartment intrinsic parallel diffusivity in NODDI may not be an appropriate setting for this study. The intrinsic parallel diffusivity used in this study was set to the commonly used value of 1.7 μm^2^·ms^−1^. However, other reports have suggested that although this value is appropriate in WM of the adult brain, a lower value may be desirable in GM (62). It cannot be ruled out that differences in the setting of the intrinsic parallel diffusivity might have altered the estimates of the NODDI parameter. Finally, it is essential to recognize that similar to the TBSS, the GBSS is not sensitive enough to detect peripheral effects that exist outside the skeleton, and the skeletal projection step may introduce biases in parameter projection (63–65).

In conclusion, the results of study suggested that the NODDI and GBSS framework can capture the detection of local GM microstructural abnormalities in adults with ASD. We found reduced NDI in the left prefrontal cortex in adults with ASD using NODDI and GBSS frameworks. Furthermore, our findings suggest the presence of disruptions of cortical cytoarchitecture in the left rostral middle frontal cortex as a neural basis for the lack of empathy in adults with ASD. The prefrontal cortex, where cortical microstructural abnormalities were suggested, is an interesting area in ASD, as recent pathological studies have reported new morphological abnormalities. More multicenter longitudinal studies with larger samples are warranted to establish evidence of GM neurodegeneration in the prefrontal cortex in ASD.

## Data availability statement

The original contributions presented in the study are included in the article/supplementary material, further inquiries can be directed to the corresponding author.

## Ethics statement

The studies involving humans were approved by the Research Ethics Committee of Juntendo University Hospital. The studies were conducted in accordance with the local legislation and institutional requirements. Written informed consent for participation was not required from the participants or the participants’ legal guardians/next of kin in accordance with the national legislation and institutional requirements. Written informed consent was obtained from the individual(s) for the publication of any potentially identifiable images or data included in this article.

## Author contributions

TA, KK, WU, CA, RI, EK, and SA contributed to the conception and design of the study. TA, WU, CA, KT, YS, RE, ZM, and EK contributed to the data collection, acquisition, and analysis of data. TA, KK, WU, CA, KT, YS, RE, ZM, RI, and SA contributed to drafting the manuscript and preparing the figures. All authors contributed to the article and approved the submitted version.

## Funding

This research was supported by Grants-in-Aid for Scientific Research of the Japan Society for the Promotion of Science (JSPS KAKENHI; Grant/Award Nos. 21K15851 and 19K17244), Japan Agency for Medical Research and Development under Grant No. JP21wm0425006, and the Juntendo Research Branding Project.

## Conflict of interest

The authors declare that the research was conducted in the absence of any commercial or financial relationships that could be construed as a potential conflict of interest.

## Publisher’s note

All claims expressed in this article are solely those of the authors and do not necessarily represent those of their affiliated organizations, or those of the publisher, the editors and the reviewers. Any product that may be evaluated in this article, or claim that may be made by its manufacturer, is not guaranteed or endorsed by the publisher.

## Footnotes

^1^https://github.com/daducci/AMICO

^2^https://github.com/arash-n/GBSS

^3^http://surfer.nmr.mgh.harvard.edu/fswiki

## Abbreviations

AQ: Autism-spectrum quotient
ATR: Anterior thalamic radiation
DTI: Diffusion tensor imaging
DWI: Diffusion-weighted images
FA: Fractional anisotropy
FWE: Family-wise error
GBSS: GM-based spatial statistics
GLM: General linear model
GM: Gray matter
ISOVF: Isotropic volume fraction
MD: Mean diffusivity
MLR: Multiple linear regression
MRI: Magnetic resonance imaging
NODDI: Neurite orientation dispersion and density imaging
ODI: Orientation dispersion index
RD: Radial diffusivity
ROI: Region-of-interest
SBCT: Surface-based cortical thickness
TA: The authors
TBSS: Tract-based spatial statistics
VBA: Voxel-based analysis
WI: Weighted images
WM: White matter
ASD: Autism-spectrum disorder
EQ: Empathizing quotient
ICV: Intracranial volume
NDI: Neurite density index
SQ: Systemizing quotient
TD: Typically developing
